# Humidex-related risk of other infectious diarrhea in Anhui, China: a multi-city study on spatial heterogeneity and socioeconomic modifiers

**DOI:** 10.1186/s12889-026-28928-3

**Published:** 2026-08-07

**Authors:** Meng Gao, Jinling Song, Jingyu Wu, Siqi Xu, Liping Gong, Yongsheng Ye, Yangguang Wang, Jian Cheng, Yinguang Fan

**Affiliations:** Department of Epidemiology and Biostatistics, School of Public Health, Anhui Medical University, Hefei, China

**Keywords:** Other Infectious Diarrhea, Humidex, Spatial Heterogeneity, Socioeconomic Factors, Distributed Lag Non-linear Model

## Abstract

**Background:**

Climate change may influence the transmission dynamics of enteric pathogens through changes in temperature, humidity, precipitation, and environmental exposure. Humid heat poses a significant threat, yet its synergistic impact on Other Infectious Diarrhea (OID) remains under-characterized. This study quantifies humid-heat-related OID risks and investigates the socioeconomic determinants driving spatial vulnerability in Anhui, China.

**Methods:**

Daily data on OID cases (*n* = 822,220) and meteorological variables were collected from 16 cities in Anhui Province (2015–2023). We employed a two-stage strategy: city-specific distributed lag non-linear models (DLNM) to estimate exposure-response associations, followed by multivariate meta-regression to evaluate geographical and socioeconomic effect modifiers. Spatial clustering was quantified using Global Moran’s *I* statistics.

**Results:**

The association between Humidex and OID was non-linear, with significant spatial clustering that intensified over time (Moran’s *I*: 0.36–0.47, *P* < 0.001). Substantial heterogeneity was observed (*I*² = 70.03%), masking marked disparities: less-developed northern cities faced significantly higher risks (e.g., Bengbu, RR = 2.08) and larger disease burdens (e.g., Suzhou, AN = 7,671) than southern counterparts. Multivariate meta-regression identified urbanization rate, GDP per capita, and latitude as the primary drivers of this regional heterogeneity.

**Conclusion:**

Humid-heat-related OID risk is structurally modulated by socioeconomic development. Urbanization and economic capacity act as critical buffers, highlighting an “adaptation deficit” in less-developed regions. Public health interventions should prioritize high-risk northern hotspots to mitigate climate-driven health inequities.

**Supplementary Information:**

The online version contains supplementary material available at https://doi.org/10.1186/s12889-026-28928-3.

## Introduction

Infectious diarrhea remains a significant public health concern and a major cause of mortality in developing countries [1]. According to the Global Burden of Disease Study 2016, diarrheal diseases are among the leading causes of death worldwide [2]. In China, Other Infectious Diarrhea (OID)—defined as infectious diarrhea excluding cholera, dysentery, typhoid, and paratyphoid fever—has consistently ranked second among Class C notifiable infectious diseases, with an average annual incidence of approximately 60.64 per 100,000 population between 1986 and 2016 [3, 4]. In recent years, the incidence of OID has been increasing, particularly among children aged ≤ 4 years, highlighting its importance as a public health challenge [5, 6].

Amid the rising burden of OID, identifying environmental determinants has become increasingly important. Climate change may influence the transmission dynamics of enteric pathogens through changes in temperature, humidity, precipitation, and human environmental exposure [7, 8]. Most epidemiological studies to date have considered ambient temperature and relative humidity as independent covariates to estimate weather-related health risks. However, this approach may inadequately reflect the combined thermal stress experienced by the human body [9, 10]. From a physiological perspective, relative humidity influences human thermal perception by modulating evaporative cooling through sweating [11], high humidity can impede heat dissipation, thereby exacerbating physiological stress even under moderate temperatures [12]. From an ecological standpoint, the interaction of heat and moisture creates favorable conditions for pathogen survival and replication: humidity prolongs the viability of desiccation-sensitive bacteria such as Campylobacter, while elevated temperatures accelerate their growth [13–15]. Accordingly, composite indices like the Humidex are theoretically more appropriate for evaluating the health risks associated with heat and humidity. Preliminary provincial-level analyses indicated that models incorporating Humidex provided a significantly more parsimonious and better fit than dual-variable models using temperature and humidity separately (*ΔQAIC* = 684.99; (Table S1). Based on these statistical results, Humidex was utilized as the primary exposure metric to capture the synergistic environmental stress relevant to OID while maintaining model stability.

Despite the utility of Humidex as a composite metric, a critical knowledge gap remains regarding the spatial heterogeneity of its health impacts. Multi-city studies, particularly those addressing mortality and non-communicable diseases, have consistently demonstrated that heat-related health risks are not evenly distributed but vary substantially across regions [16]. For OID, such spatial variation is expected to be even more pronounced, as vulnerability to enteric infections is fundamentally influenced by local adaptive capacity, which is closely associated with socioeconomic status (SES) and urban infrastructure. Rapid urbanization may exert dual and potentially opposing effects: high population density may facilitate pathogen transmission, whereas improved sanitation infrastructure (e.g., sewage treatment systems) may reduce exposure to enteric pathogens. In parallel, adaptive resources such as air conditioning may reduce vulnerability to humid-heat exposure. The limited availability of empirical evidence on socioeconomic modifiers currently hampers the identification of regions with high vulnerability, thereby constraining the development of targeted public health interventions [17]. In particular, few studies have quantitatively assessed how city-level development indicators, including GDP and urbanization rates, modify the association between humid-heat and OID [18].

This study aimed to: (1) quantify the non-linear and delayed effects of humid-heat, as measured by the Humidex, on OID incidence in 16 major cities using distributed lag non-linear models (DLNMs); (2) characterize and compare the spatial heterogeneity of humid-heat-related OID risks; and (3) assess whether regional socioeconomic factors, particularly urbanization rate and GDP, modify the association between humid-heat and OID incidence.

## Data and methods

### Study area and data collection

The overall analytical framework, including data collection and the two-stage modeling process, is illustrated in Figure S1. This study was conducted in Anhui Province, China, which lies within the climatic transition zone between the subtropical and warm temperate regions [19]. The region is characterized by a typical monsoonal climate, featuring hot and humid summers with concentrated precipitation. The 16 cities were divided into Northern (Wanbei), Central (Wanzhong), and Southern (Wannan) regions using the Huai and Yangtze Rivers as natural geographical boundaries. The Huai River, in particular, acts as a significant climatic divide: the northern region (Wanbei) belongs to a semi-humid warm temperate zone with more pronounced seasonal temperature fluctuations, while the southern regions (Wanzhong and Wannan) exhibit more humid subtropical characteristics. Daily counts of OID cases in 16 major cities were obtained from the Anhui Provincial Center for Disease Control and Prevention for the period from January 1, 2015, to December 31, 2023. Under China’s statutory infectious disease reporting system, OID is classified as a Class C notifiable disease and is defined as infectious diarrhea excluding cholera, dysentery, typhoid, and paratyphoid fever. All cases were diagnosed by local physicians following the national diagnostic criteria for infectious diarrhea (WS 271–2007) issued by the National Health Commission of China. Daily meteorological data, including mean temperature and relative humidity, were obtained from the China Meteorological Data Service Center. The meteorological datasets were strictly synchronized with the OID surveillance records on a daily basis to ensure the temporal consistency required for the time-series analysis. To capture the combined thermal stress of heat and humidity, the daily Humidex was calculated using the standard formula proposed by Masterton and Richardson [20], This comprehensive indicator integrates daily average temperature (T) and relative humidity (RH) and is defined as follows

1 $$\:Humidex=T+\frac{5}{9}\times\:[6.11\times\:{10}^{\left(\frac{7.5\times\:T}{237}+T\right)}\times\:\frac{RH}{100}-10]$$

### City-level characteristics

To examine potential effect modifiers of the association between humid-heat exposure and OID risk, city-level socioeconomic and infrastructural data were obtained from the Anhui Statistical Yearbook [21]. The multi-year averages of these indicators across the entire study period were calculated for each city to serve as stable predictors, thereby representing the structural characteristics of the study areas and minimizing the bias from annual fluctuations. The selected indicators included longitude, latitude, population density, gross domestic product (GDP), urbanization rate, green coverage rate, hospital beds per capita, and sewage pipeline coverage. The urbanization rate is defined as the proportion of the permanent urban population relative to the total population in each city. This data was obtained from the Anhui Statistical Yearbook (2015–2023). To address multicollinearity, a composite Socio-Economic Status (SES) index was synthesized via Principal Component Analysis (PCA) based on GDP, urbanization rate, hospital beds per capita, and sewage coverage. The first principal component (PC1) accounted for 63.96% of the total variance, with all four variables exhibiting positive, balanced loadings (Table S2), confirming PC1 as a robust proxy for regional development. This SES index was subsequently incorporated into the multivariate meta-regression alongside other independent predictors (e.g., population density and latitude). This refined approach ensured model parsimony and maintained all Variance Inflation Factors (VIF) below 2.5. This model was subsequently used to generate Best Linear Unbiased Predictions (BLUPs) of city-specific exposure-response relationships, providing more robust estimates for the calculation of attributable fraction (AF) and attributable number (AN) in each city.

### Spatial autocorrelation analysis

To evaluate spatial clustering with enhanced statistical power, Global Moran’s *I* statistics were calculated at the district/county level (*n* = 104) across Anhui Province. This finer resolution captures localized patterns potentially obscured at the prefecture-city level. A Queen’s contiguity spatial weight matrix was employed, and residual autocorrelation in the meta-regression was assessed via a Monte Carlo permutation procedure (999 simulations). This analysis was performed using the spdep package (version 1.4.2) in R (version 4.4.0).

### Two-stage analytic framework

#### First stage: city-specific modeling

In the first stage, a quasi-Poisson generalized linear model (GLM) combined with a DLNM was applied to estimate the non-linear and delayed effects of the Humidex on OID risk in each city [22]. The model was specified as follows:

2 $$\begin{aligned} \:\mathrm{log}\left[E\left({Y}_{t}\right)\right]=\:&\alpha\:+cb\left({Humidex}_{t}\right)+ns\left({Time}_{t}\right)+\beta\:{DOW}_{t}\\&+\delta\:{Holiday}_{t}+\sum\:_{i=1}^{2}{\gamma\:}_{i}\mathrm{log}\left({Y}_{t-i}+1\right) \end{aligned}$$

Where $\:{Y}_{t}$ represents the daily number of OID cases on day * t*; $\:\alpha\:$ refers to the model intercept; * cb* is the cross-basis function based on DLNM; * ns* is the natural cubic spline; * Humidex* represents the daily humid-heat exposure; * Time* is used to control effects of long-term trends and seasonality; * DOW* indicates the day of the week; * Holiday* denotes public holidays; $\:\mathrm{log}\left({Y}_{t-i}+1\right)$ represents the log-transformed lagged cases to control for autocorrelation; * df* is degree of freedom; $\:\beta\:$, $\:\delta\:$, and $\:\gamma\:$ are the corresponding coefficients. Based on the Akaike Information Criterion (AIC), 4 df were specified for the natural cubic splines in both the exposure and lag dimensions. Taking into account the incubation periods of enteric pathogens and findings from previous research indicating that the effects of humid-heat on infectious diarrhea are predominantly acute and short-term [9, 23], we established the maximum lag time of 14 days for this study. Additionally, a natural cubic spline with 7 *df* per year was used to control for long-term trends and seasonality. A uniform 14-day lag period was applied to all cities to ensure that the results were measured consistently and could be accurately combined in the second-stage analysis. This timeframe is long enough to capture both the immediate impact of humid-heat and any potential shift in the timing of cases that might occur over a two-week period. The empirical justification and detailed validation for incorporating these autoregressive terms are provided in the Supplementary Material (Tables S11–S12, Figures S4–S6).

#### Second stage: meta-analysis and meta-regression

In the second stage, a multivariate random-effects meta-analysis was conducted to pool the city-specific overall cumulative exposure-response associations and to explore potential sources of heterogeneity [24]. To evaluate effect modification by socioeconomic status, a meta-regression model incorporating city-level characteristics was fitted and specified as follows:

3 $$\:{\widehat{\theta\:}}_{i}\sim{N}_{k}\left({X}_{i}\beta\:,\:{S}_{i}+\varPsi\:\right)$$

Where $\:{\widehat{\theta\:}}_{i}$ represents the vector of coefficients (*k*$\:\times\:1$) for city * i* obtained from the first stage; $\:{X}_{i}$ is the row vector of city-level predictors for city * i*; $\:\beta\:$ denotes the vector of meta-regression coefficients; $\:{S}_{i}$ is the within-city covariance matrix estimated in the first stage; and $\:\varPsi\:$ indicates the between-city covariance matrix representing heterogeneity.

To account for the diverse scales and units of the socioeconomic modifiers, all city-specific covariates in $\:{X}_{i}$ were standardized using *Z*-score transformation prior to the multivariate meta-regression. This standardization facilitates the direct comparison of the relative importance of different modifiers in explaining spatial heterogeneity.

The *I*^2^ statistic and Cochran’s *Q* test were used to quantify the magnitude of heterogeneity across cities. To enhance the precision of city-specific exposure-response associations, Best Linear Unbiased Predictions (BLUPs) were derived, allowing estimates for individual cities to leverage information from the pooled distribution according to the fitted meta-regression model.

#### Estimation of attributable burden

We first derived the city-specific cumulative exposure-response curves using the BLUPs from the meta-regression model. The Relative Risk (RR) for a given humidex value was calculated by exponentiating the estimated log-relative risk, using the Minimum Morbidity Humidex (MMH) — the value associated with the lowest cumulative risk of OID — as the reference point. The MMH was used as the city-specific reference, defined as the Humidex value associated with the lowest cumulative OID risk. To quantify the burden of OID attributable to humid-heat exposure, these RR estimates were then used to calculate the AF and AN [25]. Because humid-heat exposure is largely confined to the warm season in Anhui Province, and risk estimates outside this period are more likely to be unstable due to limited exposure contrast, the attribution analysis was limited to May-October, consistent with standard practice in heat-health studies. The city-specific MMH was defined as the reference level. AF and AN were calculated using the following equations:

4 $$\:AN=\sum\:_{j=1}^{n}\frac{{RR}_{j}-1}{{RR}_{j}}\times\:{N}_{j}$$

5 $$\:\mathrm{A}\mathrm{F}=\frac{\mathrm{A}\mathrm{N}}{\sum\:_{\mathrm{j}=1}^{\mathrm{n}}{\mathrm{N}}_{\mathrm{j}}}$$

Where $\:{RR}_{j}$ is the relative risk associated with the Humidex level on day *j*, compared with the city-specific MMH. The attribution calculations were restricted to days when the Humidex exceeded the MMH and $\:{RR}_{j}$ > 1; $\:{N}_{j}$ was the daily number of OID cases; and * n* represents the total number of days in the warm season.

To characterize the delayed effects of humid heat, we extracted city-specific lag-response associations based on the BLUPs derived from the final multivariate meta-regression model. Specifically, we estimated lag-response curves (Lag 0–14 days) at an extreme humid-heat level, defined as the 90th percentile of the city-specific Humidex distribution. To represent the risk structure relative to a typical daily exposure, the city-specific median Humidex was utilized as the reference point for these lag-response estimates. A maximum lag of 14 days was selected based on the typical incubation period of enteric pathogens causing OID and consistent with prior DLNM studies examining short-incubation diseases [26, 27]. This approach allows for a comparable assessment of lag structures across cities under locally extreme heat stress.

To ensure the validity of causal inference, we performed Augmented Dickey-Fuller (ADF) tests to verify the stationarity of daily OID cases and Humidex series. Furthermore, multicollinearity was assessed using Variance Inflation Factors (VIF) in both the first-stage and second-stage models.

To evaluate the robustness of our findings, we performed sensitivity analyses by varying key model parameters and exposure metrics. First, to assess model specification robustness, we tested alternative configurations for the DLNM: (1) varying the *df* for long-term trends (6 and 8 per year, compared to the baseline of 7); (2) altering the maximum lag period (7 and 21 days, compared to the baseline of 14 days); and (3) modifying the *df* for the cross-basis function (5 *df*). Second, to validate the choice of exposure metric, we compared the risk estimates derived from the Humidex-based model with those from models using daily mean temperature (Tmean) and relative humidity (RH) separately. These comparisons were conducted to determine which metric best captured the heterogeneity of thermal stress.

### Clinical trial registration

This study is a retrospective analysis based on aggregated public health surveillance data and does not involve any healthcare interventions or human clinical trials. Therefore, clinical trial registration was not applicable. All data were analyzed at an aggregated level to ensure anonymity.

## Results

### Descriptive Statistics

The baseline temporal distribution of the 822,220 recorded cases is shown in Figure S2. Table 1 presents the descriptive statistics for OID cases and meteorological variables at the provincial level, with city-specific baseline characteristics provided in Table S3. The daily distribution of OID cases was right-skewed (Median: 11; IQR: 5–21). As illustrated in Figure S2, the time series of OID cases and Humidex exhibited distinct seasonal synchronization, with peak incidence consistently coinciding with the warm season.

**Table 1:** Descriptive statistics of daily OID cases, meteorological variables, and city-level socioeconomic indicators in 16 cities of Anhui Province, China (2015–2023)

Variable	Mean ± SD	Min	*P*_25_	Median	*P*_75_	Max
cases	15.60 ± 16.70	0	5	11	21	190
Meteorological variables						
Humidex(unitless)	20.40 ± 14.20	-13.8	7.76	20.11	32.67	48.5
Tmean(℃)	16.80 ± 9.20	-8.68	8.75	17.52	24.84	34.79
RH(%)	74.20 ± 13.10	24.55	65.41	75.4	83.98	99.75
Meteorological variables (warm season)						
Humidex (warm season) (unitless)	31.90 ± 8.24	5.94	26.09	32.59	38.47	48.5
Tmean (warm season) (℃)	24.23 ± 4.69	8.46	21.13	24.77	27.71	34.79
RH (warm season) (%)	76.38 ± 11.33	25.11	69.47	77.29	84.47	99.53
Social environmental variables						
GDP (CNY)	58,389.30 ± 23,521.50	28,918.12	37,631.53	57,789.21	65,823.82	104,468.80
Sewage pipeline coverage(km)	754.30 ± 730.50	307.55	426.03	562.25	749.54	3,370.10
density (people/km^2^)	2,813.53 ± 1,058.57	814.55	2,229.26	2,694.39	3,618.05	4,333.95
Urbanization rate(%)	57.00 ± 10.30	41.55	50.67	56.58	63.08	78.22
Total hospital beds	22,747.21 ± 13,763.14	7,905.35	13,898.23	22,467.99	24,946.74	59,759.53
Latitude (°N)	-	29.71	-	-	-	33.96
Longitude (°E)	-	115.78	-	-	-	118.76
Green Coverage Rate (%)	43.42 ± 2.05	39.24	42.3	43.27	44.96	47.63

Meteorological and case data are calculated based on daily records, while socioeconomic variables represent the annual averages across the 16 cities during the study period

*SD* Standard Deviation, ***P***_***25***_ 25th Percentile, ***P***_***75***_ 75th Percentile, *OID* Other Infectious Diarrhea, *Tmean* Mean Temperature, *RH *Relative Humidity warm season defined as May to October, *GDP *Gross Domestic Product per capita

**Fig. 1:**
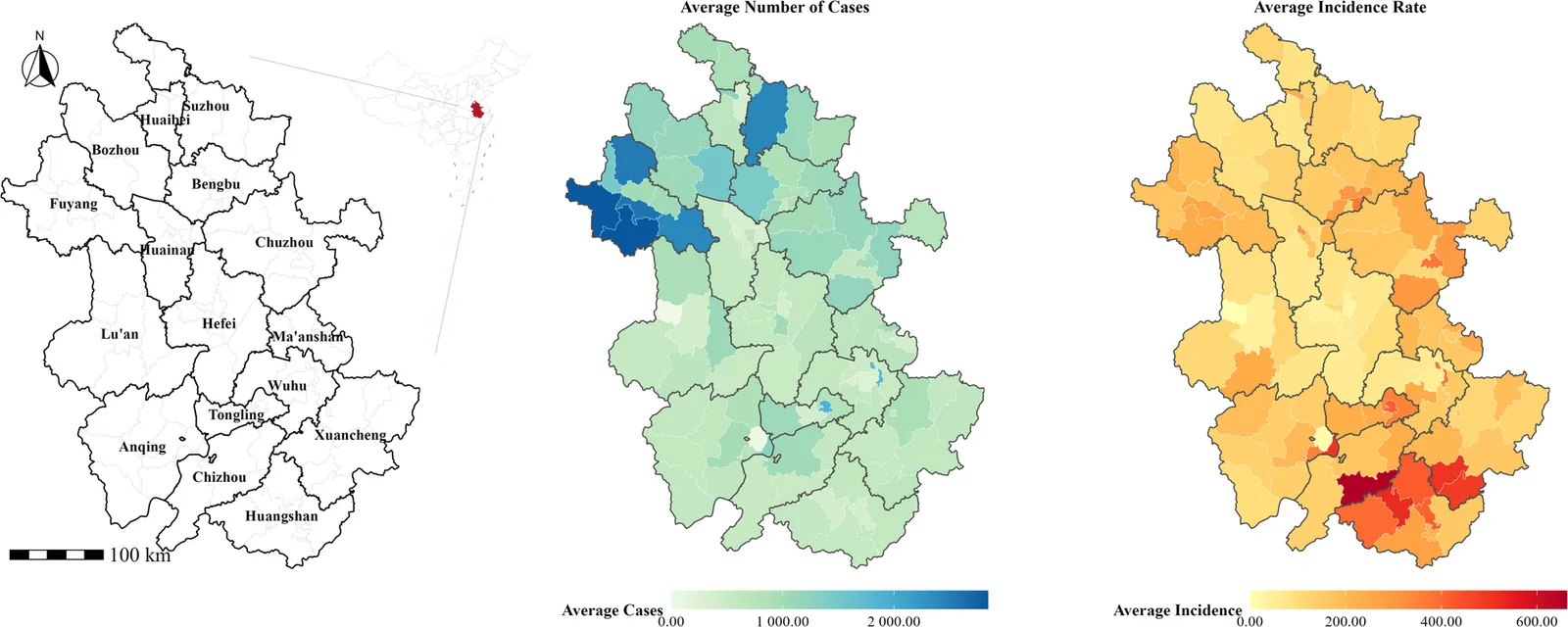
Geographic location and spatial distribution of OID in Anhui Province, China (2015–2023). **A** Geographic location of Anhui Province in China and its administrative divisions at the district/county level (*n* = 104). **B** Spatial distribution of the average annual number of OID cases, showing higher concentrations in populous northern regions. **C** Spatial distribution of the average annual OID incidence rate (per 100,000 population). The base map was obtained from the standard map service system with approval number GS (2024) 0650

The spatial patterns of absolute burden and relative risk exhibited significant geographical divergence across Anhui Province (Fig. 1). While the absolute disease burden, represented by average annual cases, was predominantly concentrated in the populous regions of Northern Anhui (Wanbei)—most notably in Fuyang and Suzhou (Fig. 1B)—the average incidence rates in these areas did not uniformly exceed those in other regions. In contrast, higher incidence rates were primarily observed in parts of Southern (Wannan) and Central Anhui (Fig. 1C), suggesting that the relative risk of OID does not strictly follow the spatial distribution of absolute case counts. While the warm-season (May to October) average Humidex exhibited a distinct south-to-north gradient (Figure S3), its spatial divergence from absolute OID cases (Fig. 1B) suggests that regional vulnerability and population density interact with meteorological exposure to shape the observed epidemic patterns.

### Spatial clustering patterns

Spatial analysis at the district/county level (*n* = 104) revealed significant and persistent clustering of OID incidence throughout the study period. The Global Moran’s *I* statistics remained consistently positive, with values ranging from 0.36 to 0.47 (all *P* < 0.001). Notably, the magnitude of this clustering exhibited an increasing temporal trend, rising from 0.37 in 2020 to a peak of 0.47 in 2023 (Table S4), suggesting an intensification of regional disparities in disease burden.

### Exposure-response relationships and regional heterogeneity of the Humidex-OID association

**Fig. 2:**
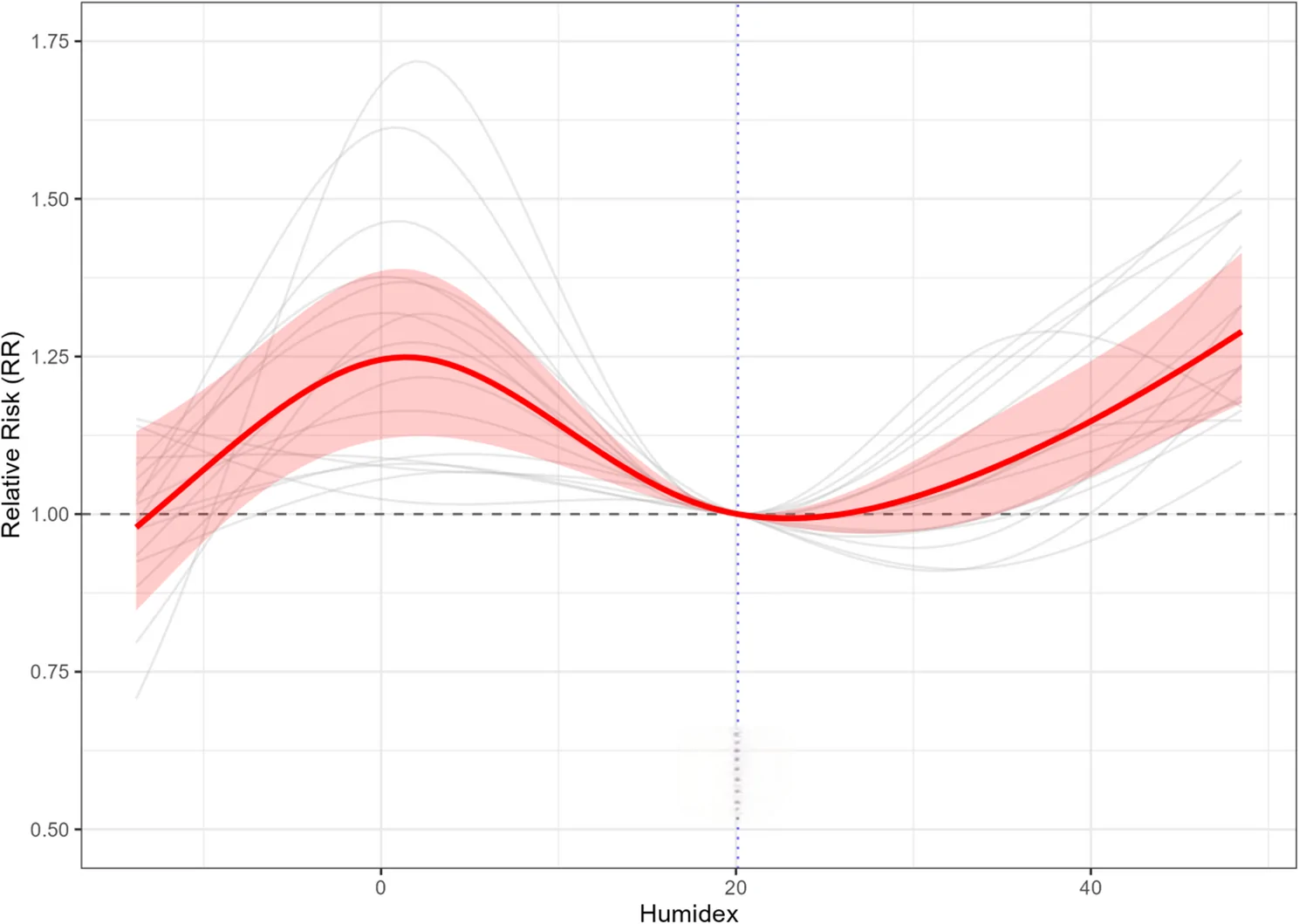
Overall cumulative exposure-response association between Humidex and OID risk in Anhui Province

The pooled cumulative exposure-response curve (Fig. 2) revealed a complex non-linear association between Humidex and OID risk at the provincial scale. Relative to the uniform reference level (set at the provincial median of 20.11 units), the risk trajectory was non-monotonic, characterized by two distinct phases of rapid escalation (reaching a peak RR of approximately 1.25 in the lower range and 1.30 at extreme intensities) interspersed with a transient decline toward the reference point. This fluctuating pattern indicates that OID risk is sensitive to both moderate and extreme humid-heat exposures. However, this aggregated profile masked substantial spatial heterogeneity, which was further explored through stratified and city-specific analyses.

**Fig. 3:**
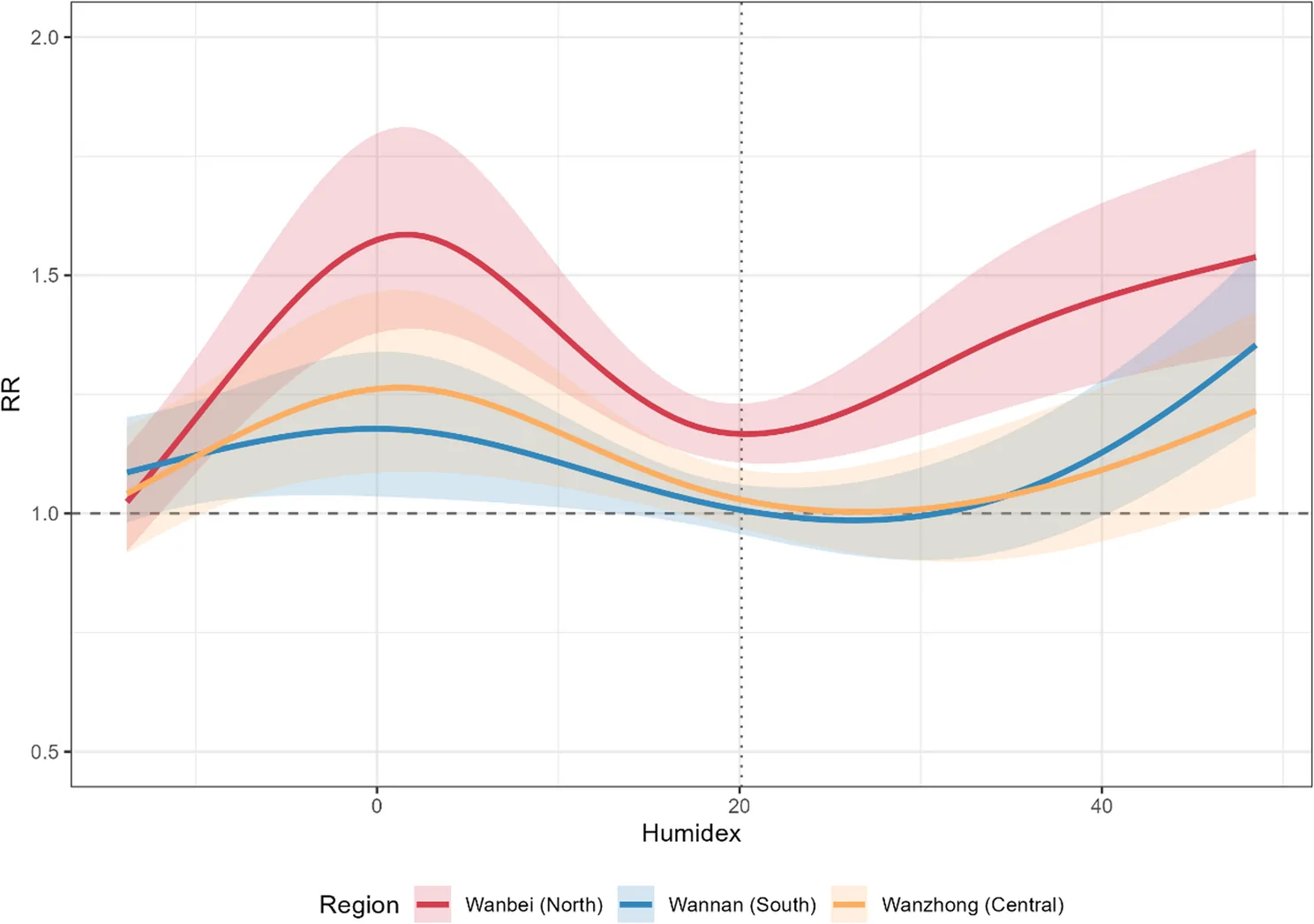
Regional average exposure-response curves between Humidex and OID risk

Stratified analysis by region demonstrated a pronounced geographic gradient (Fig. 3). The exposure-response curve for Northern Anhui (Wanbei) was the steepest, exhibiting a sharp, nearly linear surge in risk at higher Humidex intensities. This reflects a heightened sensitivity to thermal stress in the region. In contrast, Central (Wanzhong) and Southern Anhui (Wannan) displayed attenuated associations, characterized by comparatively flatter trajectories and higher thresholds for risk onset.

**Fig. 4:**
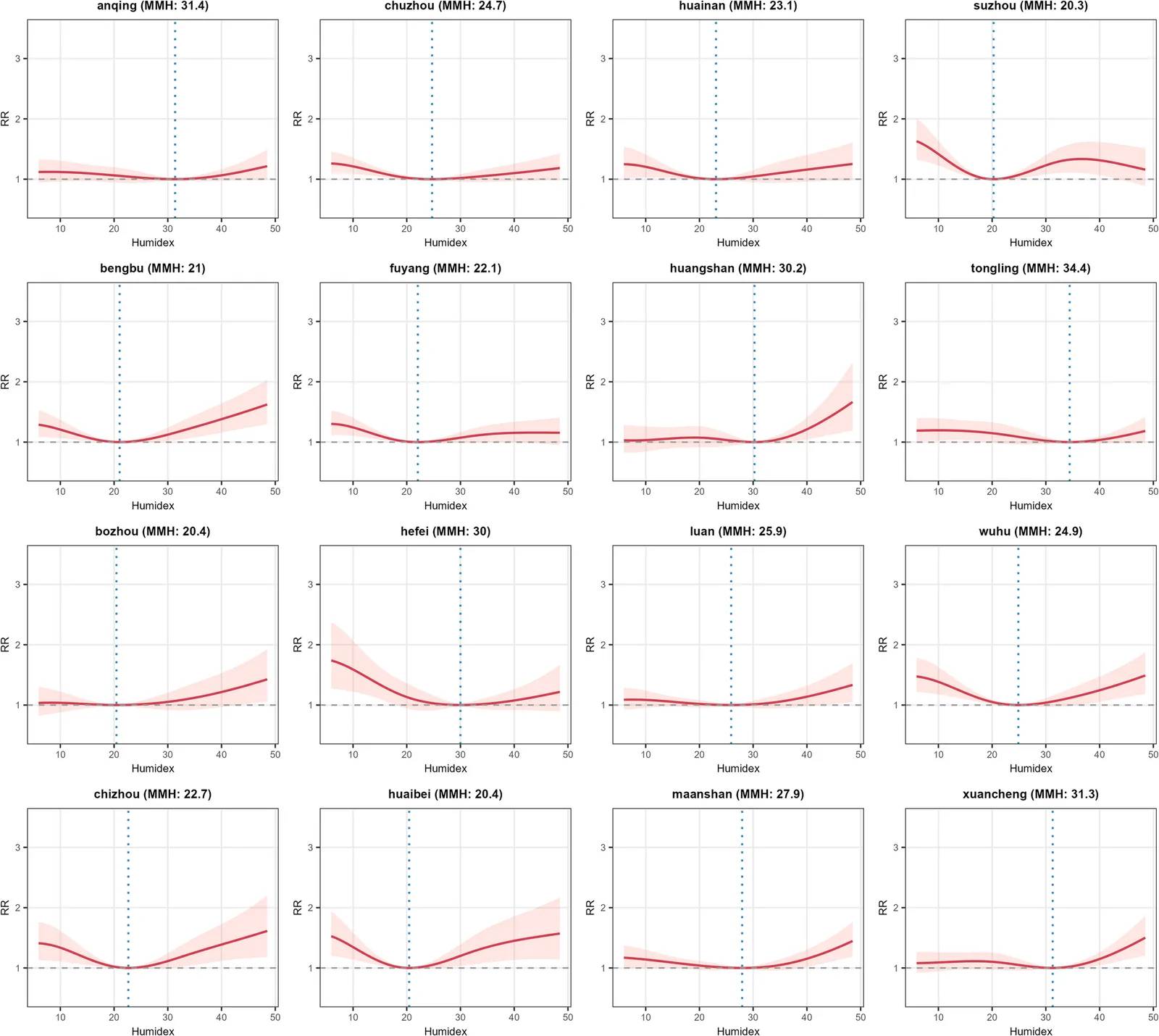
City-specific cumulative exposure-response associations between Humidex and OID risk restricted to the warm season

City-specific analysis (Fig. 4) further decomposed this heterogeneity using MMH as the local reference. The steepness of the risk curves served as a primary differentiator of vulnerability. Less-developed northern cities (e.g., Fuyang, Bozhou) showed a precipitous rise in risk immediately following their respective thresholds, suggesting high susceptibility. Conversely, socioeconomically advanced cities (e.g., Hefei) displayed flatter curves and lower effect magnitudes, potentially reflecting greater thermal resilience.

**Fig. 5:**
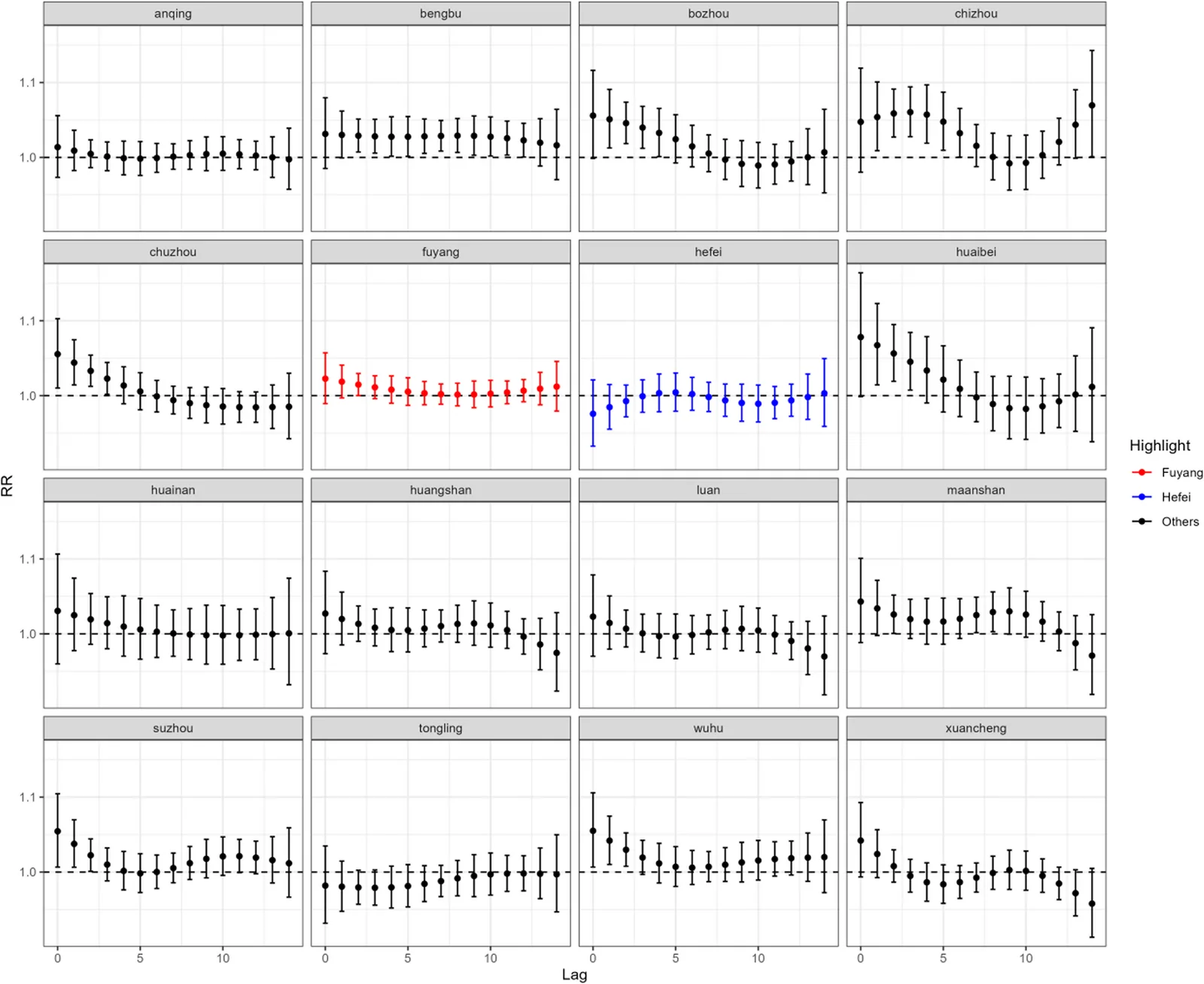
The diagram of city-specific lag-effects of specific Humidex (90th percentile) on OID in 16 cities with reference of the city-specific median

The temporal dynamics of these associations were characterized using city-specific lag-response curves under extreme humid-heat conditions (Fig. 5). Overall, the impact of humid heat on OID was predominantly acute, peaking within Lag 0–1 days. However, the persistence of these effects varied by city. In certain northern municipalities (e.g., Fuyang), elevated risks extended over longer lag periods, whereas southern cities (e.g., Hefei) showed a sharper and more rapid attenuation of risk.

### Model diagnostics and effect modification by socioeconomic and geographical factors

Diagnostic checks confirmed the robustness of our models. The ADF tests indicated that all OID series were stationary (*P* < 0.05, Table S5). In the first-stage DLNM, VIF values for Humidex ranged from 9.00 to 11.32; while relatively high, this is typical for environmental time-series data where predictors are seasonally synchronized.

**Fig. 6:**
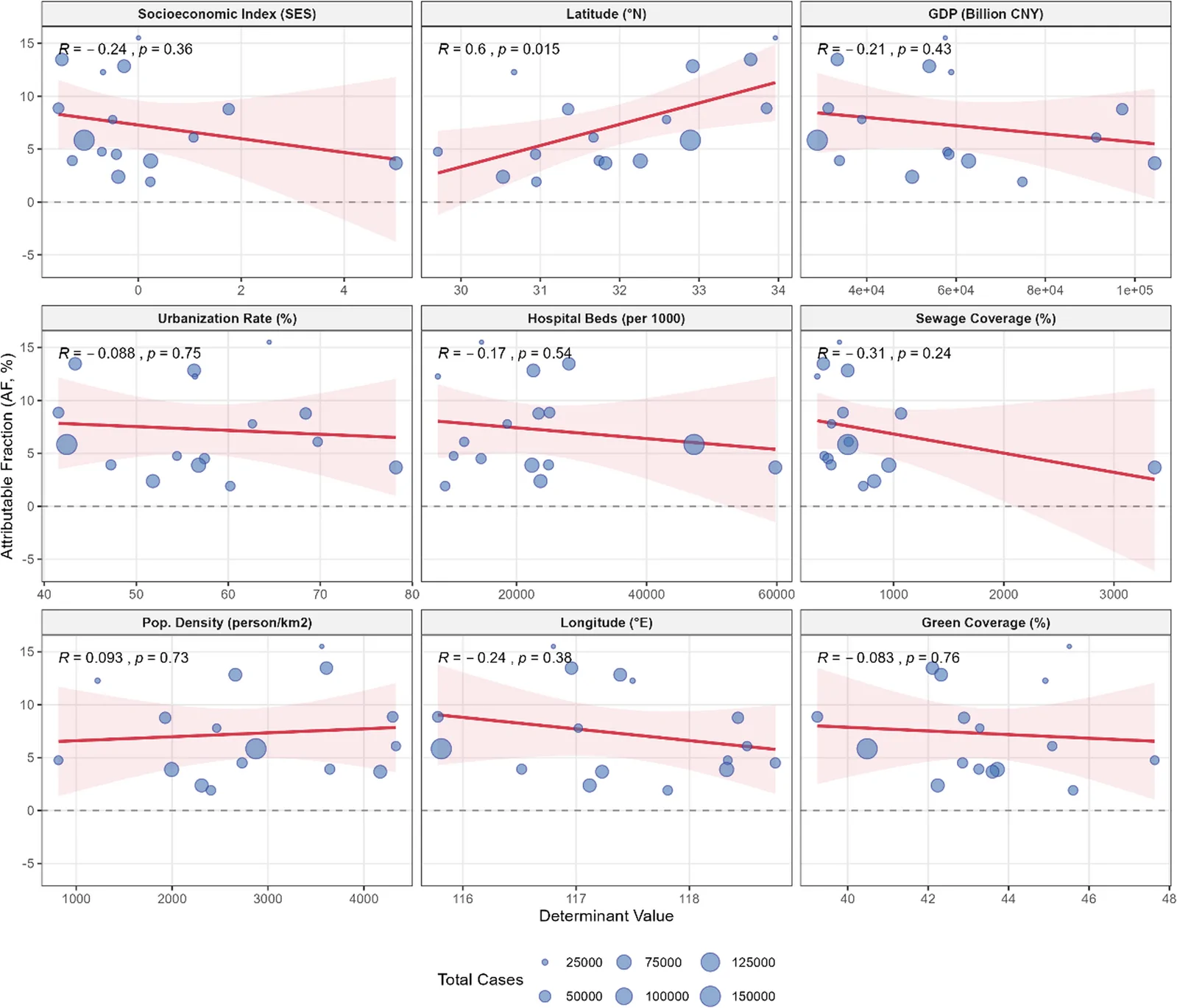
Correlations between socioeconomic/geographical characteristics and city-specific attributable fractions (AF%) of OID during the warm season

Meta-regression identified the drivers of the substantial observed heterogeneity (*I*^2^ = 70.03%, *P* < 0.05). The final multivariate model revealed that the composite SES Index and latitude were the primary significant effect modifiers (*P* < 0.05, Table 2). Notably, the SES Index—synthesized via PCA to represent integrated development—showed a significant negative association with risk estimates, suggesting that advanced socioeconomic development acts as a protective buffer. Conversely, a distinct latitudinal risk gradient was confirmed, with higher-latitude cities (Northern Anhui) exhibiting significantly elevated risks. The numerical stability of this final model was high, with all VIF values remaining below 2.5 (Table S6), effectively ruling out bias from multicollinearity among the predictors. The spatial heterogeneity and potential determinants of the OID burden during the warm season are visualized in Fig. 6. In Fig. 6, the simple correlation between the SES Index and city-specific AFs shows a weak and non-significant downward trend (*R* = -0.24, *P* = 0.36). This is largely because geographic factors confound this simple pairwise comparison. However, after controlling for geography in the multi-variable model (Table 2), the SES Index becomes statistically significant (*P* < 0.05). This indicates that the simple scatter plot fails to reveal a clear pattern due to unadjusted geographical confounding. Once regional differences are properly adjusted, higher socioeconomic development clearly reduces the humid-heat-driven OID burden. More importantly, latitude displayed a distinct and statistically significant positive trajectory (*R* = 0.60, *P* = 0.015), underscoring the significantly increased vulnerability of northern populations in Anhui Province to humid-heat exposure compared to their southern counterparts.

**Table 2:** Heterogeneity analysis and multivariate meta-regression results for potential effect modifiers of the Humidex-OID association

Factors	Cochran Q test			I^2^	LR test		Wald test			
	Q	df	*P*	(%)	Statistic	*P*	Statistic	*P*	Heterogeneity_Explained	
Intercept -only	200.22	60	< 0.05	70.03	-	-	-	-		
Socioeconomic Factors										
GDP	175.49	56	< 0.05	68.09	9.51	0.05	11.78	0.02	12.36%	*
Total hospital beds	173.61	56	< 0.05	67.86	5.21	0.27	5.71	0.22	12.99%	
Sewage pipeline coverage	190.08	56	< 0.05	70.54	8.66	0.07	9.17	0.06	5.07%	
Urbanization_rate	173.00	56	< 0.05	67.63	13.24	0.01	17.54	< 0.01	13.59%	*
SES index	173.61	56	< 0.05	67.74	11.66	0.02	15.43	< 0.01	13.29%	*
Geographical Factors										
Latitude	144.46	56	< 0.05	61.24	12.01	0.02	9.60	0.05	27.85%	*
Longitude	170.78	56	< 0.05	67.21	5.27	0.26	5.08	0.28	14.71%	
Environmental Factors										
Green Coverage Rate	177.74	56	< 0.05	68.49	5.27	0.26	4.90	0.30	11.23%	
Population density	198.44	56	< 0.05	71.78	0.69	0.95	0.62	0.96	0.89%	

The intercept-only model represents the baseline heterogeneity without predictors. Each socioeconomic factor was added individually to the meta-regression model to test its modification effect.

*I*^2^ Residual heterogeneity statistic, *LR* test Likelihood Ratio test, *df* Degrees of freedom

* indicates a statistically significant contribution to explaining heterogeneity (*P* < 0.05)

### Attributable burden in the warm season

Consistent with the risk patterns, the burden of OID attributable to humid heat exhibited marked spatial heterogeneity across Anhui Province (Table 3). Provincially, the attributable burden followed a pronounced latitudinal gradient, diminishing from north to south. The aggregate absolute burden across the 16 cities was substantial, with an estimated total of 55,807 attributable cases during the study period.

**Table 3:** Estimated AN and AF of OID cases due to humid-heat exposure during the warm season (May-October) in 16 cities

City	MMH	AN (95% CI)	AF	95% CI	
Huaibei	20.44	3,661(1,359-5,708)	15.51%	5.76%	24.19%
Suzhou	20.27	7,671(2,782 − 12,166)	13.47%	4.89%	21.36%
Bengbu	21.03	7,673(3,491 − 11,453)	12.84%	5.84%	19.16%
Chizhou	22.65	2,965(1,101-4,635)	12.27%	4.56%	19.18%
Bozhou	20.44	3,901(180-8,247)	8.86%	0.41%	18.73%
Wuhu	24.87	4,251(1,364-7,149)	8.77%	2.81%	14.74%
Huainan	23.08	2,489(0–5,395)	7.79%	0%	16.89%
Maanshan	27.94	2,210(745-3,790)	6.09%	2.05%	10.45%
Fuyang	22.06	8,767(427 − 16,960)	5.84%	0.28%	11.29%
Huangshan	30.25	1,607(426-2,763)	4.75%	1.26%	8.17%
Xuancheng	31.27	1,853(639-2,916)	4.50%	1.55%	7.09%
Luan	25.90	1,572(93 − 3,351)	3.91%	0.23%	8.34%
Chuzhou	24.71	2,789(2–6,271)	3.88%	0.28%	8.73%
Hefei	29.99	2,200(0–5,759)	3.67%	0%	9.61%
Anqing	31.36	1,485(10 − 3,254)	2.38%	0.02%	5.21%
Tongling	34.42	713(0–1,505)	1.91%	0%	4.03%

The MMH for each city was identified specifically within the observed Humidex range of the warm season (May-October) to ensure biological plausibility. The AN and AF were calculated based on daily humid-heat exposure exceeding the city-specific MMH during the warm season

*ANA* Attributable Number, *AF *Attributable Fraction, *CI *Confidence Interval

At the city level, northern cities bore the heaviest brunt of this impact. Huaibei exhibited the highest Attributable Fraction (AF) of 15.51% (95% *CI*: 5.76%, 24.19%), indicating that approximately one-seventh of warm-season OID cases in this city were associated with humid-heat exposure. Similarly, Bengbu and Suzhou faced substantial burdens, with AFs of 12.84% (95% *CI*: 5.84%, 19.16%) and 13.47% (95% *CI*: 4.89%, 21.36%), respectively. Notably, Fuyang recorded the largest absolute number of attributable cases (AN = 8,767; 95% *CI*: 427, 16,960), highlighting a critical public health challenge in this populous northern region.

Conversely, cities in the central and southern regions—characterized by higher socioeconomic development—demonstrated markedly lower attributable burdens. Hefei, the provincial capital, recorded an AF of only 3.67% (95% *CI*: 0%, 9.61%), while Tongling registered the lowest fraction province-wide at 1.91% (95% *CI*: 0%, 4.03%). This pronounced disparity further underscores the protective role of socioeconomic advancement against humid-heat impacts.

### Sensitivity analysis

Sensitivity analyses generally corroborated the robustness of the primary model specifications (Table S7). Altering the degrees of freedom for long-term trends (6–8 per year) or the cross-basis function resulted in negligible fluctuations in risk estimates (< 10% variation relative to the main model). However, the association exhibited distinct temporal sensitivity. Shortening the maximum lag period to 7 days unmasked a stronger and statistically significant risk (*RR* = 1.12, 95% *CI*: 1.01–1.23), representing a 16.7% increase in the RR estimate compared to the baseline 14-day lag. This finding provides compelling empirical evidence of the highly acute nature of the humid-heat impact on OID, which primarily clusters within the first week. Nonetheless, maintaining the uniform 14-day lag as our primary specification serves as a more rigorous and conservative baseline. While the 7-day window captures the immediate peak of the environmental shock, the extended 14-day window remains indispensable to capture the full picture of delayed secondary transmission chains and potential risk displacement (harvesting effects), thereby offering a robust estimation of the net cumulative disease burden. Conversely, extending the window to 21 days attenuated the signal (*RR* = 0.93), reinforcing the observation that humid-heat effects on OID are predominantly acute.Regarding exposure metrics, while pooled provincial estimates for both Mean Temperature (*RR* = 1.09, 95% *CI*: 0.94–1.27) and Humidex (*RR* = 0.96, 95% *CI*: 0.83–1.11) lacked statistical significance at the extended lag, city-specific comparisons highlighted the superior sensitivity of Humidex in high-risk regions (Table S8). For instance, in northern cities such as Bengbu, the risk estimate derived from the Humidex model (*RR* = 2.08) was notably higher than that obtained from the temperature-only model (*RR* = 1.59), suggesting that Humidex better captures the synergistic stress of heat and moisture in vulnerable areas.

## Discussion

To our knowledge, this is one of the first multi-city studies to systematically quantify the non-linear and delayed effects of humid heat on OID in this climatic transition zone. Our analysis identified Humidex as a critical environmental driver of OID in Anhui, revealing a complex non-linear association at the provincial level that masks profound spatial heterogeneity. We observed an intensifying spatial clustering of risk over time—confirmed by rising Global Moran’s *I* indices—which highlights widening regional health inequalities. Notably, the statistical validity of our modeling framework was confirmed by the spatial independence of the residuals (Monte Carlo Moran’s *I* = -0.126, *P* = 0.647), suggesting that these spatial structures were effectively accounted for by the included modifiers. Regarding temporal dynamics, humid-heat exerted an acute impact on OID, with peak risks occurring within the first 7 days. The subsequent risk attenuation dropped below the baseline in specific cities (Fig. 5), suggesting a potential ‘harvesting effect’ (risk displacement). Essentially, thermal stress may accelerate disease onset among vulnerable individuals. This temporal pattern highlights the acute nature of the humid-heat impact. Indeed, the environmental effect peaks intensely within a compressed 7-day lag window, yielding a stronger statistical signal. Therefore, a 14-day lag window offers a more cautious and conservative appraisal of the net cumulative risk. This choice ensures the full capture of post-acute compensatory reductions.

A key finding of this study is the significant modification effect of socioeconomic development. There was a distinct latitudinal risk gradient: less-developed northern regions exhibited steep, precipitous exposure-response curves and high attributable burdens, whereas socioeconomically advanced cities displayed flatter trajectories. This latitudinal gradient is deeply rooted in the regional climatic configuration of Anhui Province. As a pivotal transition zone between North and South China, the province is bisected by the Huai River, which serves as the traditional climatic boundary between the warm-temperate and subtropical zones [19]. The northern region experiences a temperate monsoon climate with lower baseline temperatures and humidity, leading to a population with lower physiological acclimatization to sustained humid-heat compared to the humid subtropical south. Consequently, when exposed to similar humid-heat intensities during the warm season, northern populations exhibit a lower adaptation threshold and higher biological sensitivity. This stark contrast provides empirical evidence supporting the “protective buffering” role of modernization and infrastructure against climate-sensitive diseases [16, 28–31]. The positive association observed between Humidex and OID risk aligns with established biological plausibility regarding pathogen survival and host susceptibility [29, 32–36]. Our findings critically extend this understanding. We demonstrate that these baseline biological risks are not uniformly distributed but are structurally modulated by local adaptive capacities, making socioeconomic status a decisive determinant of vulnerability. Furthermore, the credibility of our causal inference is bolstered by both temporal and statistical evidence. Humidex exposure precedes the health outcome, satisfying a fundamental criterion for causal inference (Hill’s criteria) [37]. This causal link is further supported by rigorous diagnostic checks (ADF and VIF) that effectively ruled out the possibility of spurious correlations [22].

We further examined susceptibility across demographic groups (Table S9). Intriguingly, the age-specific results diverged from the traditional assumption that physiological vulnerability (e.g., immature or declining immunity in children and the elderly) dictates risk [29, 32]. Instead, we observed higher risk point estimates among working-age adults (16–60 years) compared to older adults (> 61 years) and young children (0–5 years), although the interaction test did not reach statistical significance (*P*_for interaction_ > 0.05). This pattern suggests that, in the context of OID transmission in Anhui, behavioral exposure pathways may outweigh physiological vulnerability. During periods of humid heat, working-age adults exhibit higher occupational mobility and are more likely to engage in outdoor dining behaviors (e.g., consumption of street food, barbecue, or cold beverages), which significantly increases exposure vectors for foodborne and waterborne pathogens [35, 36]. Conversely, children and older adults in Chinese households often benefit from a “behavioral shielding effect.” These groups are more likely to remain indoors in climate-controlled environments and consume home-prepared meals during extreme weather, thereby reducing their exposure to environmental heat stress and high-risk dietary sources [33, 34]. This interpretation aligns with prior epidemiological evidence in China, highlighting the critical role of behavioral patterns in shaping the seasonal dynamics of enteric infections.

The identification of urbanization rate and GDP as significant effect modifiers suggests that socioeconomic development confers resilience through multi-layered pathways.

First, at the community level, modern infrastructure acts as a critical barrier to pathogen transmission. Higher urbanization rates are intrinsically linked to robust sanitation systems, including extensive sewage networks and treated water supplies [18, 38, 39], These infrastructural defenses effectively intercept fecal-oral transmission routes, severing the link between climatic suitability for pathogen growth and actual human infection, even under favorable weather conditions. Consequently, the attenuated risks observed in developed cities likely reflect this “sanitation shield”.

Second, at the household level, economic prosperity enhances adaptive capacity. Higher disposable income facilitates access to protective resources: widespread refrigeration prevents food spoilage during humid-heat events, while air conditioning mitigates the physiological thermal stress that can compromise host immunity [40, 41]. Conversely, the steep, monotonically increasing risk trajectories in Northern Anhui highlight a profound “adaptation deficit”. Characterized by a predominantly agriculture-based economy and lower urbanization, this region lacks both the infrastructural buffering of modern cities and the household-level resources to mitigate exposure. This suggests that without the protective benefits of modernization, populations remain highly sensitive to environmental thermal shifts, leading to exacerbated vulnerability to climate-sensitive enteric infections [42].

These findings advocate for a paradigm shift from uniform intervention strategies toward precision public health approaches that explicitly account for spatial heterogeneity. While current climate adaptation protocols often apply blanket vulnerability assessments across broad regions [13, 43], our results warrant a tiered response framework tailored to local sensitivity profiles and disease burdens.

The divergence in exposure-response patterns—characterized by precipitous risk escalations in less-developed northern regions versus resilient trajectories in urban centers—suggests that intervention thresholds should be dynamically adjusted. specifically, lower activation thresholds for health alerts are necessary in high-susceptibility areas. Furthermore, the disproportionate attributable burden—with over one-quarter of warm-season cases in northern cities (e.g., Suzhou, Fuyang) linked to humid heat, compared to less than 5% in Hefei—highlights a critical imperative for equity-driven resource allocation. Consequently, preventative resources—such as enhanced water quality surveillance, rigorous food safety inspections, and targeted educational campaigns—must be prioritized for these identified northern hotspots during the warm season (May-October) to maximize the cost-effectiveness of health protection.

Several limitations should be acknowledged. First, regarding exposure assessment, the choice of Humidex reflects a trade-off. While it effectively captures the combined stress of temperature and humidity, it does not account for other thermal factors such as solar radiation and wind speed, potentially leading to non-differential exposure misclassification. Second, the unavailability of pathogen-specific data precluded the analysis of etiological heterogeneity, preventing us from distinguishing whether specific pathogens (e.g., viral vs. bacterial) drive the observed heat sensitivity. Thirdly, despite controlling for temporal trends and seasonality, residual confounding from unmeasured time-varying factors (e.g., changes in hygiene habits or diet) cannot be entirely ruled out. Fourth, while this study identifies key socioeconomic modifiers, it does not fully address potential nonlinear threshold effects or the compound interactions between climatic and socioeconomic drivers. Future research could utilize emerging approaches, such as spatial machine learning or Geographically Explainable AI (Geo-XAI), to better capture these complex interdependencies and quantify the fine-grained determinants of spatial health risk patterns [44, 45]. Fifth, the stratified analyses by age and sex may have limited sensitivity for detecting interaction effects due to the second-stage sample size (*N* = 16). While the variations in point estimates provide exploratory insights into potential demographic disparities, these results should be interpreted with caution. Finally, as an ecological study, the findings represent population-level associations and should be interpreted with caution regarding individual-level risks (ecological fallacy). However, the comprehensive multi-city design and rigorous control of spatial-temporal patterns substantiate the internal validity and regional representativeness of the observed heterogeneity.

## Conclusion

Our study provides robust evidence characterizing the non-linear and synergistic impact of humid heat on OID in Anhui Province, confirming Humidex as a sensitive predictor of enteric infection risk. Quantitatively, the burden of OID attributable to humid-heat exposure varied significantly across the province, with AFs ranging from 1.91% to 15.51% across the 16 cities, resulting in a total of 55,807 attributable cases (AN) during the study period. Crucially, we unveiled a distinct spatial heterogeneity masked by provincial averages: the “North-High, South-Low” risk gradient is significantly driven by regional socioeconomic disparities rather than meteorological variation alone. We identified urbanization rate and economic growth as critical “buffers” that confer resilience against climate-sensitive diseases, highlighting the heightened vulnerability of less-developed, agriculture-intensive regions. Consequently, current uniform adaptation strategies may be insufficient. We advocate for a paradigm shift toward precision public health interventions that prioritize resource allocation—such as sanitation infrastructure and health education—to the identified northern hotspots. Addressing these socioeconomic determinants is imperative for mitigating climate-driven health inequities and achieving sustainable disease control in a warming world.

## Supplementary Information

Supplementary Material 1.

## Data Availability

Daily data on other infectious diarrheal diseases were collected from the Infectious Disease Report Cards of the Anhui Provincial Centre for Disease Control and Prevention. Daily meteorological data for mean temperature and relative humidity were obtained from the China Meteorological Data Service Center (http://data.cma.cn/). Geographic, demographic, economic, and infrastructural indicators were obtained from the Anhui Provincial Statistical Yearbook. The datasets generated and/or analysed during the current study are not publicly available due to data privacy restrictions of the disease surveillance system but are available from the corresponding author on reasonable request.
