# Supplementary material for: Humidex-related risk of other infectious diarrhea in Anhui, China: a multi-city study on spatial heterogeneity and socioeconomic modifiers
Source: BMC Public Health. 2026 Aug 7;26:3196. doi: 10.1186/s12889-026-28928-3 (PMC13733127; doi:10.1186/s12889-026-28928-3)
Supplement: Supplementary file 1 [file 12889_2026_28928_MOESM1_ESM.docx]

Figure S1. Detailed analytical framework of the study.

Note: The workflow diagram describes the two-stage approach: (Stage I) city-specific distributed lag non-linear modeling for 16 cities in Anhui Province, and (Stage II) multivariate meta-analysis incorporating a synthesized Socio-Economic Status (SES) index. It also highlights the attribution analysis process for calculating the disease burden related to humid-heat.

Figure S2. Daily distribution of Other Infectious Diarrhea (OID) cases and Humidex in Anhui Province, China (2015–2023).

Figure S3. Spatial distribution of mean Humidex during the warm season (May–October) in Anhui Province.

Figure S4. Comparison of Residual Partial Autocorrelation Function (PACF) Plots Between Basic and Autoregressive Models.

Note: This figure provides a diagnostic comparison of temporal dependence in the model residuals for all 16 study cities.

Group A (Red): The basic GLM-DLNM model without autoregressive terms. Significant residual autocorrelation is observed at lag 1 and lag 2 (spikes exceeding the blue dashed lines), indicating that the environmental variables alone cannot fully account for the temporal correlation in OID incidence.

Group B (Green): The final model incorporating 1-day and 2-day log-transformed lagged cases ($\sum_{i=1}^{2} \gamma_{i}\log\left( Y_{t-i}+1 \right)$).

Figure S5. City-specific Humidex-RR curves comparison between Model A (without autoregression) and Model B (primary model, with autoregression) with 95% confidence intervals across 16 cities during the warm season (May–October).

Figure S6. Provincial pooled Humidex-RR curves comparison between Model A and Model B with 95% confidence intervals during the warm season (May–October).

Table S1. Comparison of model fit statistics (QAIC) at the provincial aggregate level.

| **Model Exposure Metric** | **QAIC** | **ΔQAIC (vs. Humidex)** |
| --- | --- | --- |
| Humidex | 65,168.06 | Reference |
| Tmean | 64,392.21 | -775.85 |
| RH | 64,307.64 | -860.42 |
| Dual-variable (Tmean + RH) | 65,853.05 | 684.99 |

Note: A lower *QAIC* indicates a better model fit. While single-variable models show lower values due to simpler parameters, the Humidex model is preferred over the dual-variable configuration for its high parsimony and over single variables for its biological completeness.

Table S2. Results of the Principal Component Analysis (PCA) for the Socio-Economic Status (SES) Index.

| **Variable / Metric** | **PC1 (SES Index)** | **PC2** | **PC3** | **PC4** |
| --- | --- | --- | --- | --- |
| Loadings (Eigenvectors) |  |  |  |  |
| Gross Domestic Product (GDP) | 0.548 | -0.373 | 0.658 | 0.357 |
| Urbanization Rate | 0.539 | -0.392 | -0.736 | 0.12 |
| Hospital Beds per Capita | 0.295 | 0.773 | -0.107 | 0.552 |
| Sewage Pipeline Coverage | 0.568 | 0.331 | 0.119 | -0.744 |
| Importance of Components |  |  |  |  |
| Standard Deviation | 1.599 | 1.126 | 0.333 | 0.251 |
| Proportion of Variance (%) | 63.96% | 31.69% | 2.78% | 1.57% |
| Cumulative Proportion (%) | 63.96% | 95.65% | 98.43% | 100% |

Note: SES, Socio-Economic Status; GDP, Gross Domestic Product; PC, Principal Component.

Table S3. Baseline demographic, meteorological, and socioeconomic characteristics of the 16 cities in Anhui Province (2015–2023).

| **City** | **Region** | **Total Cases** | **Daily Cases (Median, IQR)** | **Humidex (Mean ± SD)** | **GDP (Billion)** | **Urbanization (%)** | **Density (pop/km2)** | **Sewage Coverage (%)** | **Hospital Beds (/1000)** | **SES Index** |
| --- | --- | --- | --- | --- | --- | --- | --- | --- | --- | --- |
| fuyang | Wanbei (North) | 150163 | 38 (17-64) | 19.8 (14.4) | 28918.1 | 42.4 | 2874 | 584.4 | 47269.27 | -1.88 |
| chuzhou | Wanzhong (Central) | 71878 | 18 (9-31) | 19.9 (14.3) | 62813 | 56.8 | 1994 | 956.8 | 22355 | 0.12 |
| anqing | Wanzhong (Central) | 62493 | 18 (9-27) | 21.4 (13.9) | 50156.2 | 51.8 | 2308 | 823.6 | 23682.08 | -0.6 |
| hefei | Wanzhong (Central) | 59960 | 14 (6-29) | 20.9 (14.2) | 104468.8 | 78.2 | 4170 | 3370.1 | 59759.53 | 2.84 |
| bengbu | Wanbei (North) | 59782 | 13 (6-28) | 19.7 (14.5) | 53993.9 | 56.3 | 2659 | 583.6 | 22580.98 | -0.18 |
| suzhou | Wanbei (North) | 56949 | 12 (4-27) | 18.9 (14.6) | 33397.5 | 43.4 | 3609 | 362.9 | 28033.56 | -1.68 |
| wuhu | Wannan (South) | 48487 | 13 (8-20) | 21.7 (14.1) | 97191.5 | 68.4 | 1927 | 1068.1 | 23383.67 | 1.95 |
| bozhou | Wanbei (North) | 44043 | 10 (4-19) | 19.4 (14.5) | 31386.2 | 41.5 | 4300 | 540.9 | 25063.52 | -1.86 |
| xuancheng | Wannan (South) | 41152 | 11 (6-18) | 20.7 (13.7) | 58414.3 | 57.4 | 2729 | 405.2 | 14548.97 | 0.03 |
| luan | Wanzhong (Central) | 40189 | 11 (6-17) | 19.8 (14) | 33889.4 | 47.2 | 3645 | 433 | 22666.65 | -1.4 |
| tongling | Wannan (South) | 37324 | 9 (3-17) | 21.9 (14.1) | 74856.3 | 60.2 | 2404 | 724.9 | 9018.1 | 0.72 |
| maanshan | Wannan (South) | 36279 | 10 (5-15) | 21.2 (14.2) | 91371.9 | 69.7 | 4334 | 592.2 | 11946 | 1.86 |
| huangshan | Wannan (South) | 33806 | 9 (5-14) | 20.7 (13.2) | 58002.5 | 54.4 | 815 | 370.4 | 10320.96 | -0.18 |
| huainan | Wanbei (North) | 31950 | 6 (2-15) | 20.2 (14.4) | 38878.9 | 62.6 | 2464 | 436.2 | 18565.43 | -0.2 |
| chizhou | Wannan (South) | 24162 | 6 (3-10) | 21.2 (13.7) | 58913.8 | 56.4 | 1222 | 307.6 | 7905.35 | -0.02 |
| huaibei | Wanbei (North) | 23603 | 6 (2-11) | 18.9 (14.6) | 57576 | 64.5 | 3561 | 509 | 14615.09 | 0.49 |

Note: Data Presentation: Meteorological variables (Humidex) and daily cases are presented as Mean ± Standard Deviation (SD) or Median (Interquartile Range, IQR). Socioeconomic indicators represent the annual averages over the study period. Abbreviations: OID: Other Infectious Diarrhea; GDP: Gross Domestic Product; SES Index: Socioeconomic Status Index (a composite index synthesized via PCA based on GDP, urbanization rate, hospital beds per capita, and sewage pipeline coverage). Region: The province is divided into Northern Anhui (Wanbei), Central Anhui (Wanzhong), and Southern Anhui (Wannan).

Table S4 Global autocorrelation analysis of OID in Anhui province, 2015-2023

| **Year** | **Moran's *I*** | ***Z*-value** | ***P-value*** |
| --- | --- | --- | --- |
| 2015 | 0.40 | 6.97 | ＜0.001 |
| 2016 | 0.40 | 7.11 | ＜0.001 |
| 2017 | 0.42 | 7.57 | ＜0.001 |
| 2018 | 0.38 | 6.66 | ＜0.001 |
| 2019 | 0.36 | 6.35 | ＜0.001 |
| 2020 | 0.37 | 6.47 | ＜0.001 |
| 2021 | 0.43 | 7.54 | ＜0.001 |
| 2022 | 0.45 | 7.84 | ＜0.001 |
| 2023 | 0.47 | 8.11 | ＜0.001 |

Table S5 Augmented Dickey-Fuller (ADF) test results for daily OID cases and Humidex series across 16 cities in Anhui Province.

| **City** | **Variable** | **Lag** | **ADF Statistic** | **P-value** | **Conclusion** |
| --- | --- | --- | --- | --- | --- |
| anqing | OID Cases | 14 | -4.38 | <0.05 | Stationary |
|  | Humidex | 14 | -2.72 | 0.27 | Non-stationary |
| bengbu | OID Cases | 14 | -3.44 | <0.05 | Stationary |
|  | Humidex | 14 | -2.61 | 0.32 | Non-stationary |
| bozhou | OID Cases | 14 | -3.82 | <0.05 | Stationary |
|  | Humidex | 14 | -2.61 | 0.32 | Non-stationary |
| chizhou | OID Cases | 14 | -5.98 | <0.05 | Stationary |
|  | Humidex | 14 | -2.71 | 0.28 | Non-stationary |
| chuzhou | OID Cases | 14 | -4.76 | <0.05 | Stationary |
|  | Humidex | 14 | -2.61 | 0.32 | Non-stationary |
| fuyang | OID Cases | 14 | -3.57 | <0.05 | Stationary |
|  | Humidex | 14 | -2.64 | 0.31 | Non-stationary |
| hefei | OID Cases | 14 | -3.92 | <0.05 | Stationary |
|  | Humidex | 14 | -2.74 | 0.26 | Non-stationary |
| huaibei | OID Cases | 14 | -5.29 | <0.05 | Stationary |
|  | Humidex | 14 | -2.58 | 0.33 | Non-stationary |
| huainan | OID Cases | 14 | -4.44 | <0.05 | Stationary |
|  | Humidex | 14 | -2.63 | 0.31 | Non-stationary |
| huangshan | OID Cases | 14 | -5.21 | <0.05 | Stationary |
|  | Humidex | 14 | -2.79 | 0.24 | Non-stationary |
| luan | OID Cases | 14 | -5.19 | <0.05 | Stationary |
|  | Humidex | 14 | -2.68 | 0.29 | Non-stationary |
| maanshan | OID Cases | 14 | -4.11 | <0.05 | Stationary |
|  | Humidex | 14 | -2.62 | 0.32 | Non-stationary |
| suzhou | OID Cases | 14 | -3.8 | <0.05 | Stationary |
|  | Humidex | 14 | -2.56 | 0.34 | Non-stationary |
| tongling | OID Cases | 14 | -4.77 | <0.05 | Stationary |
|  | Humidex | 14 | -2.65 | 0.30 | Non-stationary |
| wuhu | OID Cases | 14 | -4.42 | <0.05 | Stationary |
|  | Humidex | 14 | -2.76 | 0.26 | Non-stationary |
| xuancheng | OID Cases | 14 | -4.23 | <0.05 | Stationary |
|  | Humidex | 14 | -2.7 | 0.28 | Non-stationary |

Note: *P* < 0.05 indicates the series is stationary. For Humidex series that exhibit non-stationarity (due to strong seasonality), the long-term trends and seasonal cycles were further controlled using natural cubic splines with 7 *df* /year in the DLNM framework.

Table S6 Multicollinearity Diagnostics for the Final Meta-Regression Model

| **Predictor Variable** | **Variance Inflation Factor (VIF)** |
| --- | --- |
| SES Index (PC1) | 1.41 |
| Latitude (°N) | 2.34 |
| Longitude (°E) | 2.1 |
| Population Density | 2.09 |
| Green Coverage Rate | 1.73 |
| Mean VIF | 1.93 |

Note: Multicollinearity was assessed via Variance Inflation Factors (VIF). Additionally, a Moran’s I test was performed on the model residuals using a Monte Carlo permutation procedure (999 simulations) to check for spatial autocorrelation; the result (Moran’s *I* = -0.126, *P* = 0.647) confirmed no significant spatial dependency remained in the final model.

Table S7. Sensitivity analysis of the pooled association between Humidex and OID risk under different model specifications in Anhui Province (2015–2023).

| **Scenario** | **RR_P95** | **Change_Pct** |
| --- | --- | --- |
| Baseline (df=7/yr, Lag=14) | 0.96 (0.83-1.11) | 0.00% |
| Max Lag = 7 | 1.12 (1.01-1.23) | 16.80% |
| Time df = 6/yr | 0.95 (0.84-1.08) | -0.50% |
| Time df = 8/yr | 0.88 (0.74-1.04) | -8.20% |
| Max Lag = 21 | 0.93 (0.74-1.16) | -3.10% |
| Basis df = 5 | 1.00 (0.85-1.17) | 4.40% |

Note: RR: Relative Risk; CI: Confidence Interval; df: degrees of freedom. The "Main Model" refers to the baseline model specification used in the primary analysis (4 df for cross-basis functions, 7 df/year for time trends, and a maximum lag of 14 days). Models 1–3 examine the robustness of the results by varying the degrees of freedom for long-term trends (6–8 df/year). Models 4–6 assess the impact of altering the maximum lag period (7, 14, and 21 days). Risk estimates are pooled at the provincial level.

Table S8. Comparison of city-specific risk estimates (RR) for OID derived from models using Humidex, Mean Temperature, and Relative Humidity.

| **City** | **RR_Tmean** | **RR_RH** | **RR_Humidex** |
| --- | --- | --- | --- |
| Anqing | 1.21 (1.01-1.44)* | 0.79 (0.70-0.88) | 1.14 (0.95-1.39) |
| Bengbu | 1.59 (1.31-1.92)* | 0.87 (0.76-0.99) | 2.08 (1.67-2.59)* |
| Bozhou | 1.30 (1.03-1.63)* | 0.93 (0.80-1.09) | 1.71 (1.31-2.23)* |
| Chizhou | 2.17 (1.64-2.87)* | 0.68 (0.55-0.84) | 2.41 (1.78-3.26)* |
| Chuzhou | 1.31 (1.09-1.59)* | 0.98 (0.87-1.12) | 1.26 (1.02-1.55)* |
| Fuyang | 1.26 (1.10-1.45)* | 0.93 (0.84-1.03) | 1.37 (1.17-1.61)* |
| Hefei | 0.94 (0.77-1.14) | 1.07 (0.93-1.23) | 0.92 (0.74-1.16) |
| Huaibei | 1.66 (1.22-2.25)* | 0.76 (0.63-0.93) | 1.73 (1.22-2.47)* |
| Huainan | 1.09 (0.81-1.46) | 0.77 (0.63-0.95) | 1.24 (0.88-1.73) |
| Huangshan | 1.26 (1.02-1.55)* | 0.77 (0.65-0.90) | 1.39 (1.10-1.75)* |
| Luan | 1.31 (1.04-1.63)* | 0.74 (0.62-0.87) | 1.24 (0.96-1.61) |
| Maanshan | 1.55 (1.23-1.96)* | 1.05 (0.91-1.22) | 1.60 (1.25-2.06)* |
| Suzhou | 1.30 (1.08-1.57)* | 0.86 (0.76-0.98) | 1.43 (1.15-1.77)* |
| Tongling | 0.75 (0.60-0.93) | 0.76 (0.64-0.90) | 0.74 (0.58-0.94) |
| Wuhu | 1.51 (1.24-1.85)* | 0.80 (0.70-0.91) | 1.74 (1.40-2.18)* |
| Xuancheng | 1.02 (0.84-1.25) | 0.87 (0.74-1.02) | 1.09 (0.87-1.35) |

Note: * indicates a statistically significant (*P* < 0.05)

Table S9. Subgroup analysis of the cumulative relative risk (RR) of OID associated with high humid-heat (90th percentile) stratified by gender and age.

| **Group** | **Subgroup** | **Total Cases** | **RR_P90** | ***P* for Interaction** |
| --- | --- | --- | --- | --- |
| Gender |  |  |  |  |
|  | Male | 400444 | 1.43 (1.22-1.66) | Ref |
|  | Female | 421776 | 1.48 (1.31-1.68) | 0.695 |
| Age Group |  |  |  |  |
|  | Age 0-5 | 91705 | 1.39 (1.07-1.80) | 0.319 |
|  | Age 6-15 | 64590 | 1.28 (0.98-1.67) | 0.137 |
|  | Age 16-30 | 163931 | 1.63 (1.36-1.97) | Ref |
|  | Age 31-45 | 144976 | 1.53 (1.26-1.86) | 0.626 |
|  | Age 46-60 | 174762 | 1.73 (1.32-2.26) | 0.746 |
|  | Age 61+ | 182256 | 1.32 (1.03-1.68) | 0.165 |

Note: Settings: The Relative Risk (RR) was estimated at the 90th percentile of the Humidex distribution relative to the provincial median, accumulated over a 0–4 day lag period to capture acute effects.Ref: Reference category for interaction tests.P-value: Calculated using a *Z*-test to assess the statistical significance of the difference in risk estimates between subgroups compared to the reference group (Male for gender; Age 16–30 for age groups).Interpretation: Although working-age adults (16–60 years) showed higher risk estimates compared to children and the elderly, the differences were not statistically significant (*P* > 0.05).

Table S10. Mean AIC values of the first-stage models for 16 cities across different *df* combinations.

| ***df* (Predictor \ Lag)** | 3 | 4 | 5 | 6 |
| --- | --- | --- | --- | --- |
| 3 | 19807.02 | 19807.77 | 19807.35 | 19807.61 |
| 4 | 19781.28 | **19781.54*** | 19782.55 | 19782.27 |
| 5 | 19771.31 | 19771.08 | 19772.47 | 19770.95 |
| 6 | 19768.16 | 19768.8 | 19770.48 | 19768.36 |

Note: The values are the average AIC scores across all study cities. The consistent use of 4 *df* across all cities ensures the stability of the subsequent meta-regression and spatial analysis.

Table S11. Sensitivity analysis of the warm-season (May–October) attributable burden of other infectious diarrhea (OID) under different model specifications (Model A vs. Model B) across 16 cities in Anhui Province.

| City | MMH | Model A | | | | Model B | | | | Relative Change |
| --- | --- | --- | --- | --- | --- | --- | --- | --- | --- | --- |
|  |  | *AN* (95% CI) | *AF* | 95% *CI* | | *AN* (95% CI) | *AF* | 95% *CI* | |  |
| Huaibei | 20.44 | 5,355(2,533-7,732) | 22.69% | 10.73% | 32.76% | 3,661(1,359-5,708) | 15.51% | 5.76% | 24.19% | -31.64% |
| Suzhou | 20.27 | 11,739(5,464-17,243) | 20.61% | 9.59% | 30.28% | 7,671(2,782-12,166) | 13.47% | 4.89% | 21.36% | -34.66% |
| Bengbu | 21.03 | 13,096(8,031-17,590) | 21.91% | 13.43% | 29.42% | 7,673(3,491-11,453) | 12.84% | 5.84% | 19.16% | -41.41% |
| Chizhou | 22.65 | 5,416(2,968-7,301) | 22.42% | 12.28% | 30.22% | 2,965(1,101-4,635) | 12.27% | 4.56% | 19.18% | -45.27% |
| Bozhou | 20.44 | 8,586(564-15,942) | 19.49% | 1.28% | 36.20% | 3,901(180-8,247) | 8.86% | 0.41% | 18.73% | -54.56% |
| Wuhu | 24.87 | 7,114(2,667-10,676) | 14.67% | 5.50% | 22.02% | 4,251(1,364-7,149) | 8.77% | 2.81% | 14.74% | -40.24% |
| Huainan | 23.08 | 3,780(188-7,442) | 11.83% | 1% | 23.29% | 2,489(0-5,395) | 7.79% | 0% | 16.89% | -34.15% |
| Maanshan | 27.94 | 3,946(933-6,899) | 10.88% | 2.57% | 19.02% | 2,210(745-3,790) | 6.09% | 2.05% | 10.45% | -43.99% |
| Fuyang | 22.06 | 19,751(8,672-30,084) | 13.15% | 5.78% | 20.03% | 8,767(427-16,960) | 5.84% | 0.28% | 11.29% | -55.61% |
| Huangshan | 30.25 | 4,336(117-9,640) | 12.83% | 0.35% | 28.52% | 1,607(426-2,763) | 4.75% | 1.26% | 8.17% | -62.94% |
| Xuancheng | 31.27 | 2,908(1,599-4,243) | 7.07% | 3.88% | 10.31% | 1,853(639-2,916) | 4.50% | 1.55% | 7.09% | -36.26% |
| Luan | 25.9 | 2,683(530-4,871) | 6.67% | 1.32% | 12.12% | 1,572(93-3,351) | 3.91% | 0.23% | 8.34% | -41.40% |
| Chuzhou | 24.71 | 5,932(543-10,828) | 8.25% | 0.76% | 15.06% | 2,789(2-6,271) | 3.88% | 0.28% | 8.73% | -52.98% |
| Hefei | 29.99 | 3,178(0-7,525) | 5.30% | 0% | 12.55% | 2,200(0-5,759) | 3.67% | 0% | 9.61% | -30.77% |
| Anqing | 31.36 | 2,656(3-6,088) | 4.25% | 0.01% | 9.74% | 1,485(10-3,254) | 2.38% | 0.02% | 5.21% | -44.06% |
| Tongling | 34.42 | 977(163-1,780) | 2.62% | 0% | 4.77% | 713(0-1,505) | 1.91% | 0% | 4.03% | -27.02% |
| Pooled (Anhui) | 20.10 | 101,452(34,977-165,883) | 12.34% | 4.25% | 20.17% | 55,808(12,618-101,322) | 6.79% | 1.53% | 12.32% | -44.99% |

Note: *AN*, attributable number; *AF*, attributable fraction; CI, confidence interval; MMH, minimum morbidity Humidex.

Model A excludes autoregressive terms.

Model B (primary model) adjusts for *Log-Lag*1 and *Log-Lag*2 of daily cases to control for epidemic momentum.

For both models, the city-specific MMH was identified within the observed warm-season Humidex range to ensure biological plausibility. The *AN* and *AF* were calculated based on daily exposure exceeding the city-specific MMH.

$$Relative Change(\%)=\frac{\text{AF}_{\text{Model B}}\text{-}\text{AF}_{\text{Model A}}}{\text{AF}_{\text{Model A}}}$$

Table S12. Sensitivity analysis of cumulative relative risks (*RR*s) at the city-specific 90th percentile (*P*_90_) Humidex level under different model specifications (Model A vs. Model B) across 16 cities during the warm season (May–October).

| City | Humidex | Model A | Model B |
| --- | --- | --- | --- |
|  | *P*_90_ | *RR* | *RR* |
| Anqing | 42.61 | 1.18 (0.96, 1.44) | 1.10 (0.98, 1.25) |
| Bengbu | 42.58 | 1.96 (1.50, 2.57) | 1.45 (1.20, 1.75) |
| Bozhou | 42.03 | 1.64 (1.06, 2.56) | 1.26 (0.99, 1.61) |
| Chizhou | 41.91 | 2.13 (1.52, 2.99) | 1.44 (1.14, 1.82) |
| Chuzhou | 42.37 | 1.29 (1.04, 1.59) | 1.12 (0.97, 1.30) |
| Fuyang | 42.37 | 1.42 (1.12, 1.78) | 1.16 (0.99, 1.35) |
| Hefei | 43.08 | 1.20 (0.90, 1.60) | 1.12 (0.91, 1.38) |
| Huaibei | 41.74 | 1.90 (1.32, 2.75) | 1.48 (1.14, 1.91) |
| Huainan | 42.82 | 1.33 (1.00, 1.76) | 1.19 (0.97, 1.46) |
| Huangshan | 40.27 | 1.53 (0.96, 2.41) | 1.22 (1.05, 1.43) |
| Luan | 41.50 | 1.34 (1.08, 1.66) | 1.17 (0.99, 1.37) |
| Maanshan | 43.33 | 1.49 (1.15, 1.92) | 1.25 (1.08, 1.45) |
| Suzhou | 41.75 | 1.50 (1.09, 2.05) | 1.29 (1.04, 1.60) |
| Tongling | 43.41 | 1.13 (1.01, 1.27) | 1.09 (0.98, 1.20) |
| Wuhu | 43.37 | 1.67 (1.27, 2.20) | 1.34 (1.11, 1.61) |
| Xuancheng | 41.49 | 1.36 (1.18, 1.57) | 1.20 (1.07, 1.34) |
| Pooled (Anhui) | 44.05 | 1.41 (1.21, 1.64) | 1.20 (1.09, 1.32) |
| City | Humidex | Model A | Model B |
|  | *P*_90_ | *RR* | *RR* |
| anqing | 42.61 | 1.18 (0.96, 1.44) | 1.10 (0.98, 1.25) |
| bengbu | 42.58 | 1.96 (1.50, 2.57) | 1.45 (1.20, 1.75) |
| bozhou | 42.03 | 1.64 (1.06, 2.56) | 1.26 (0.99, 1.61) |
| chizhou | 41.91 | 2.13 (1.52, 2.99) | 1.44 (1.14, 1.82) |
| chuzhou | 42.37 | 1.29 (1.04, 1.59) | 1.12 (0.97, 1.30) |
| fuyang | 42.37 | 1.42 (1.12, 1.78) | 1.16 (0.99, 1.35) |
| hefei | 43.08 | 1.20 (0.90, 1.60) | 1.12 (0.91, 1.38) |
| huaibei | 41.74 | 1.90 (1.32, 2.75) | 1.48 (1.14, 1.91) |
| huainan | 42.82 | 1.33 (1.00, 1.76) | 1.19 (0.97, 1.46) |
| huangshan | 40.27 | 1.53 (0.96, 2.41) | 1.22 (1.05, 1.43) |
| luan | 41.50 | 1.34 (1.08, 1.66) | 1.17 (0.99, 1.37) |
| maanshan | 43.33 | 1.49 (1.15, 1.92) | 1.25 (1.08, 1.45) |
| suzhou | 41.75 | 1.50 (1.09, 2.05) | 1.29 (1.04, 1.60) |
| tongling | 43.41 | 1.13 (1.01, 1.27) | 1.09 (0.98, 1.20) |
| wuhu | 43.37 | 1.67 (1.27, 2.20) | 1.34 (1.11, 1.61) |
| xuancheng | 41.49 | 1.36 (1.18, 1.57) | 1.20 (1.07, 1.34) |
| Pooled (Anhui) | 44.05 | 1.41 (1.21, 1.64) | 1.20 (1.09, 1.32) |

Note: *RR*, cumulative relative risk; CI, confidence interval; MMH, minimum morbidity Humidex.

Model A excludes autoregressive terms.

Model B (primary model) adjusts for *Log-Lag*1 and *Log-Lag*2 of daily cases to control for epidemic momentum.

For both model specifications, the cumulative *RR*s at the city-specific 90th percentile (*P*_90_) Humidex were calculated relative to the respective city-specific MMH identified within the warm season (May–October) to ensure biological plausibility. The provincial pooled estimates represent the pooled RR predicted at the provincial average levels of socioeconomic status (SES Index) and latitude, centered at the provincial median Humidex (20.1°C).
